# Quality Improvement Initiatives Need Rigorous Evaluation: The Case of Pressure Ulcers

**DOI:** 10.1177/1062860616666672

**Published:** 2016-08-30

**Authors:** Richard F. Averill, John S. Hughes, Richard L. Fuller, Norbert I. Goldfield

**Affiliations:** 1 3M Health Information Systems, Wallingford, CT; 2 Yale University School of Medicine, New Haven, CT

**Keywords:** Partnership for Patients, quality measurement, pressure ulcers, hospital-acquired complications

## Abstract

The Partnership for Patients (PfP) and the Agency for Healthcare Research and Quality (AHRQ) have reported a 23.5% decline in hospital-acquired pressure ulcers (HAPU) over 4 years resulting in a cumulative cost savings of more than $10 billion and 49 000 averted deaths, claiming that this significant decline may have been spurred in part by Medicare payment incentives associated with severe (stage 3 or 4) HAPUs. Hospitals with a high rate of severe HAPUs have a payment penalty imposed, creating a financial disincentive to report severe HAPUs, possibly contributing to the magnitude of the reported decline. Despite the financial disincentive to report, the number of severe HAPUs found in claims data over the corresponding 4-year period did not decline but instead remained unchanged. The results from claims data, combined with some flaws in estimating HAPUs, call into question the validity of the decline in HAPUs reported by PfP and AHRQ.

In September 2015, the Partnership for Patients (PfP),^1^ a public-private initiative led by the Centers for Medicare & Medicaid Services (CMS) received $1.1 billion in Affordable Care Act funding to continue its work in reducing preventable hospital-acquired conditions and readmissions.^2^ Since 2011, PfP and the Agency for Healthcare Research and Quality (AHRQ) have together published a national scorecard on rates of hospital-acquired conditions. In December 2015, the latest scorecard reported a 17% reduction in the total number of hospital-acquired conditions under surveillance, resulting in a cumulative cost savings of $19.8 billion, and 87 000 deaths averted. The largest single contributor to these improvements was a reduction in the number of hospital-acquired pressure ulcers (HAPUs), from 1.32 million in the baseline year of 2010 to 1.01 million in 2014, an estimated reduction of 310 000 fewer HAPUs in 2014 than would have been expected—a 23.5% decline. The cumulative reduction in HAPUs across the 4-year time period was estimated to result in cost savings of more than $10 billion, and 49 000 averted deaths.

In support of these significant declines in a relatively short time period, PfP and AHRQ explained that,

Although the precise causes of the decline in patient harm are not fully understood, the increase in safety has occurred during a period of concerted attention by hospitals throughout the country to reduce adverse events. This effort has been spurred in part by Medicare payment incentives and catalyzed by the US Department of Health and Human Services Partnership for Patients (PfP) initiative.^3^

If these dramatic improvements are accurate, then it is certainly reasonable to assume that the Medicare payment incentives aimed at lowering inpatient complications “spurred” some of the reported reductions in HAPUs. The purpose of this study is to use claims data to examine whether these estimates of HAPU reductions and the associated cost savings are in fact reasonable and accurate.

## Medicare Payment Incentives Related to HAPUs

Pressure ulcers are a localized injury to the skin and/or underlying tissue caused by pressure on the skin (sometimes referred to as bed sores). In administrative claims data, pressure ulcers are reported as stage 1 through 4 (*International Classification of Disease, Ninth Revision, Clinical Modification* [ICD-9-CM] codes 70721-70724, respectively) or, more often, without a specified stage (ICD-9-CM codes 70700-70709, 70720, 70725). Stage 3 (full thickness tissue loss) and stage 4 (full thickness tissue loss with exposed bone, tendons, or muscle) are the most severe pressure ulcers. With the implementation of Medicare Severity–Diagnosis Related Groups (MS-DRGs) in 2008, stage 3 and stage 4 pressure ulcers are considered to be a major complication or comorbidity. The presence of a secondary diagnosis that is classified as a major complication or comorbidity will often significantly increase payment under MS-DRGs. Beginning in January of 2008, hospitals were required to report in their administrative claims data whether or not each diagnosis was present on admission (POA). The POA designation allowed for the identification of hospital-acquired complications, thereby enabling the implementation of 2 policies affecting the payment of HAPUs:

- The Deficit Reduction Act of 2005 requires that certain secondary diagnoses that occur after admission (and therefore are not POA) be classified as hospital-acquired conditions and be excluded from Medicare MS-DRG assignment, resulting in lower payment for some patients.^4^ Stage 3 and stage 4 HAPUs are among the diagnoses classified as a hospital-acquired condition.
- Beginning in fiscal year (FY) 2015, the Accountable Care Act of 2010 established the Hospital-Acquired Condition Reduction Program (HACRP) that requires that the 25% of hospitals with the poorest performance on hospital-acquired complications have their Medicare payments reduced by 1%.^5^ Stage 3 and stage 4 pressure ulcers that were not POA (HAPUs) are included as complications under HACRP and have a major financial impact on the determination of the HACRP payment penalty.

The exclusion of hospital-acquired conditions from MS-DRG assignment was implemented in FY 2009. The HACRP payment penalties, although not implemented until FY 2015, are computed based on a hospital’s performance from June 2011 through December 2013. Thus, hospitals were aware well in advance that their performance in prior years would determine the FY 2015 HACRP payment penalties. The combination of HACRP penalties and hospital-acquired condition–associated payment reductions can have a substantial impact on Medicare payment.

Between 2010 and 2014, hospitals had a strong incentive to report stage 3 and stage 4 pressure ulcers that were POA, because they would be counted as a major complication or comorbidity and would increase MS-DRG payment. Conversely, there was no payment incentive to report stage 3 and stage 4 pressure ulcers that were not POA (a HAPU) because they would not increase MS-DRG payment. In addition, because the data from 2011 and 2013 would be used to compute the initial FY 2015 HACRP payment penalties, there was a financial disincentive to report stage 3 and stage 4 HAPUs because they could result in a HACRP payment penalty. The Medicare payment incentives related to the reporting of pressure ulcers can be summarized as follows:

- There is neither a financial incentive nor disincentive to report stage 1 and stage 2 pressure ulcers and pressure ulcers without a specified stage.
- There is a financial incentive to report stage 3 and stage 4 pressure ulcers that are POA.
- There is a financial disincentive to report stage 3 and stage 4 pressure ulcers that are not POA (HAPUs).

The 23.5% decline in HAPUs reported by AHRQ and PfP encompassed HAPUs across all stages. The observed rate of stage 3 and stage 4 HAPUs would be expected to show at least that amount of decline because of the lack of financial incentive to report them.

## Analysis of Claims Data

The Medicare Provider Analysis and Review inpatient hospital data for FYs 2010 to 2014 (October 1, 2009, through September 30, 2014), based on computerized hospital claims data, was used to calculate the observed rates of stage 3 and stage 4 HAPUs. As shown in Table 1, the observed rate of stage 3 and stage 4 HAPUs did not decline as expected but was essentially flat from 2010 through 2014. If there had been a 23.5% decline in stage 3 and stage 4 HAPUs, the 21 993 stage 3 and stage 4 HAPUs in 2010 would have decreased by 5168 to 16 825 in 2014. In light of the financial disincentive to report stage 3 and stage 4 HAPUs, it is difficult to reconcile a lack of any actual decline in the stage 3 and stage 4 HAPUs during the 4-year period in which AHRQ and PfP reported a dramatic decline in HAPUs across all stages. In contrast, the number of stage 1, stage 2, and unstaged HAPUs increased from 83 545 in 2010 to 132 062 (a rate increase from 0.57% to 0.88%).

**Table 1.** Stage 3 and Stage 4 HAPU Counts and Rates, 2010 to 2014.

	2010	2011	2012	2013	2014
All patient count	14 558 611	14 850 733	14 475 157	14 943 198	14 924 970
HAPU count	21 993	21 054	23 518	24 168	22 513
HAPU rate	0.15%	0.14%	0.16%	0.16%	0.15%

Abbreviation: HAPU, hospital-acquired pressure ulcer.

Source: MedPAR inpatient hospital data for October 1, 2009, through September 30, 2014.

On the other hand, the number of POA stage 3 and stage 4 pressure ulcers increased steadily from 139 991 in 2010 to 159 342 in 2014, consistent with hospitals’ financial incentive to report as many stage 3 and stage 4 pressure ulcers that were POA as possible in order to increase MS-DRG payments.

In response to the financial disincentives to record stage 3 and stage 4 HAPUs, hospitals could have coded improperly in 3 ways: (1) by shifting coding for stage 3 and stage 4 HAPUs from not POA to POA, or (2) shifting coding of stage 3 and stage 4 HAPUs to codes with no financial disincentive—stages 1, 2, or to ulcer codes without a stage, or (3) at the extreme, by simply failing to code HAPUs at all. In fact, any such improper coding would have constituted a false claim with potential audit and legal consequences and there is no reason to believe that any of these coding shifts occurred to any substantial degree. If hospitals were systematically shifting the coding of stage 3 and stage 4 pressure ulcers to POA or to lower stage ulcer codes, or failing to code them at all, it would have caused a decline in the number of stage 3 and stage 4 HAPUs, which was not observed in the Medicare data. Thus, despite the financial disincentive to report stage 3 and stage 4 HAPUs, their numbers did not actually decline.

## Discussion

The estimated 23.5% decline in HAPUs and the associated cost savings of more than $10 billion between 2010 and 2014 were derived from the Medicare Patient Safety Monitoring System (MPSMS) chart reviews of a limited subset of Medicare patients varying between 18 000 and 33 000 patients in a given year.^6^ It could be argued that chart reviews produce more accurate data on HAPUs than is reported in claims data. Although there is some merit to that argument for HAPUs that do not impact payment (stage 1, stage 2, and stage unspecified HAPUs), it is unlikely to be true for stage 3 and stage 4 HAPUs that directly impact payment. If the decline in stage 3 and stage 4 HAPUs derived from the MPSMS chart reviews was accurate, the lack of an actual decline in the claims data would imply that hospitals are systematically overreporting stage 3 and stage 4 HAPUs. Overreporting of stage 3 and stage 4 HAPUs by hospitals seems very unlikely in light of the financial disincentive to report them. As a result, the decline in HAPU rates reported by AHRQ and PfP are at least counterintuitive and at best questionable.

The rate of HAPUs for Medicare patients, derived from the MPSMS reviews, was used to estimate the number of HAPUs in the overall populations even though the HAPU rate for patients younger than age 65 is much lower than for patients ages 65 and older. Previous studies have found that only 30% of HAPUs occur in the population younger than age 65.^7^ Simply recalculating to accommodate for these differences in age-related rates would reduce the HAPU rate for the overall population by almost half. Indeed, the authors of the AHRQ and PfP study admitted that “it is possible that the ratios we estimated with those data are not correct for all patients.”^8^

Similarly the estimated $17 286 cost of a HAPU used in AHRQ and PfP estimates was based on a study of stage 3 and stage 4 HAPUs but applied to all HAPUs regardless of stage, thereby including the less costly stage 1, stage 2, and non-staged HAPUs.^9^ Because stage 3 and stage 4 ulcers make up at most 25% of all HAPUs, an inflated cost estimate was applied to the large majority of HAPU cases.^7^ The combined effect of using an inflated cost estimate applied to an inflated number of HAPUs means that the AHRQ estimated cost savings are dramatically overstated. Indeed, if the decline in HAPUs was based on claims data instead of the MPSMS chart reviews, the estimated savings associated with stage 3 and stage 4 HAPUs would be virtually zero. This conclusion is consistent with a study done by Mathematica Policy Research, which found that estimates of HAPU reductions using other data sources were minimal or nonexistent.^10^

Perhaps most troubling is that the results reported by AHRQ and PfP could be used as the basis for future policy initiatives under the assumption that substantial improvements in quality and efficiency have actually been achieved. The results reported by AHRQ and PfP have generated significant press coverage, creating an unjustified sense of confidence in the effectiveness of the PfP program.^11^ The substantial investment of more than $1.1 billion made by CMS in the PfP program certainly merits a thorough and valid evaluation. The fact that the reported results are so inconsistent with the claims data and at odds with the expectations generated by the existing payment incentives requires explanation. Furthermore, the questionable assumptions made in the process of extrapolating Medicare results to the general population needs scrutiny and correction. The authors agree with others who have called for a more considered and transparent framework within which to assess the contributions of the PfP initiative and the hospital engagement networks.^12^

## Conclusions

The 23.5% decrease in HAPUs reported by AHRQ and PfP cannot be verified using the Medicare claims database. The estimated 23.5% decline in HAPUs and the associated cost savings of more than $10 billion are based on questionable assumptions used for extrapolating Medicare results to the general population. Indeed, based on claims data, the estimated cost savings are virtually zero. A thorough and valid evaluation of the AHRQ and PfP reported results is needed before using the results as the basis for future policy decisions.
